# Matron's Department

**Published:** 1905-09-23

**Authors:** 


### Editor's Letter-Box.

### MATRON'S DEPARTMENT.

Sir,—May I venture to ask whether, in estimating the amount of work done by nurses in different hospitals, you have calculated the varying amount of help given by wardmaids? In some hospitals these do a great deal of the rough work, in others very little. It is therefore not quite correct to estimate the work accomplished by the nurses by simply calculating the amount of beds per nurse and leaving out the wardmaid factor.

Yours faithfully,
E. L. C. Eden.

[The point raised is a very interesting one, and the supposition that a proportionately small nursing staff needs to be supplemented by extra wardmaids at first seems sound and logical. But the tables given in "Burdett's Annual for 1905" (pages 132-5) supply no evidence in favour of it. On the contrary, it will be seen that the number of the wardmaids and scrubbers swells almost in proportion to the increased size of the nursing staff. It need not be rashly inferred that a large nursing staff "makes work"; it is more reasonable to conclude that the same causes which demand an increase in the number of nurses lead to pressure on the wardmaids' time. In the London Hospital, for instance, the average number of beds works out at two for each nurse, and 13 for each wardmaid. At Guy's it is 2·1 for each nurse, and 10 for each wardmaid. Whereas at St. Mary's, King's College, and University College Hospital, where the size of nursing staff is appreciably smaller, the average number of beds to the wardmaid is 23. There are at the present day no general hospitals of any reputation in which what could be described as "rough work" devolves upon the nurse. Even where there are no wardmaids, as at the Royal Free, the Seamen's, and many provincial hospitals, the heavy general ward work is done as a matter of course by scrubbers or servants. But in nearly all hospitals, however lavishly supplied with wardmaids, all that relates to the individual patient falls to the nurse's share, whether menial or not, and in addition she will often be called upon to perform supplementary dusting, sweeping, polishing, or clearing up. The extent to which she learns to rely on the wardmaid for help in the performance of these duties is largely a question of habit, or to put it more plainly, of organisation.—Ed. The Hospital.]

